# Supplementary material for: Ribosome•RelA structures reveal the mechanism of stringent response activation
Source: eLife. 2016 Jul 19;5:e17029. doi: 10.7554/eLife.17029 (PMC4974054; doi:10.7554/eLife.17029)
Supplement: Figure 1—source data 1. — DOI: http://dx.doi.org/10.7554/eLife.17029.003 [file elife-17029-fig1-data1.doc]

**Figure 1-source data 1: Structure I-IV map resolution and refinement statistics*.**

|  | **Particles** | **Resolution**  **(FSC=0.143)** | **CC^a^** | **Real-space R-factor** | **RMS deviations^b^** | | |
| --- | --- | --- | --- | --- | --- | --- | --- |
|  |  |  |  |  | **Bonds (Å)** | | **Angles (°)** |
| **Structure I** | 76158 | 3.9 Å | 0.83 | 0.17 | 0.007 | 0.9 | |
| **Structure II** | 46935 | 4.1 Å | 0.82 | 0.19 | 0.01 | 1.0 | |
| **Structure III** | 77862 | 3.9 Å | 0.85 | 0.16 | 0.007 | 1.0 | |
| **Structure IV** | 57430 | 3.9 Å | 0.83 | 0.18 | 0.009 | 1.1 | |

^a^ CC, resolution-dependent correlation coefficient as defined in (Chapman, 1995).

^b^ RMS (root-mean-square) deviations from ideal covalent bond lengths and angles (Engh and Huber, 1991).

* The statistics are shown for the refined structures, in the absence of the central and N-terminal domains of RelA in Structure I (excluding aa 1-595), and in the absence of the N-terminal domains of RelA in Structures II-IV (excluding aa 1-403).
