# Supplementary material for: Ribosome•RelA structures reveal the mechanism of stringent response activation
Source: eLife. 2016 Jul 19;5:e17029. doi: 10.7554/eLife.17029 (PMC4974054; doi:10.7554/eLife.17029)
Supplement: Figure 6—source data 1. — DOI: http://dx.doi.org/10.7554/eLife.17029.020 [file elife-17029-fig6-data1.doc]

| ***Region*** | **Distance^a^ (RMSD, all-atom), Å** | | |
| --- | --- | --- | --- |
|  | **II to III** | **III to IV** | **II to IV** |
| ***50S subunit***, used to align 70S ribosomes  (23S rRNA, excluding L1 and L11 stalks) | 0.688 | 0.637 | 0.673 |
| ***Body, 30S central region***  (nt 580-920 of 16S rRNA) | 0.738 | 1.12 | 1.31 |
| ***Head***  (nt 960-1400 of 16S rRNA) | 0.842 | 1.29 | 1.42 |
| ***Shoulder, near the 30S center***  (h18, nt 510-540, 16S rRNA) | 1.19 | **2.00** | **2.92** |
| ***Shoulder,* periphery**  (h16, nt 400-440) | 1.11 | **4.06** | **4.88** |

**Figure 6-source data 1**. **Distances between Structures II, III and IV, reflecting movement of the 30S shoulder domain from Structures II to III to IV, relative to the head and body of the 30S subunit.**

^a^ Root-mean-square differences (RMSD) that are more than 3 X RMSD for the 23S rRNA, are shown in bold. Superposition of Structures II and III on Structure IV was obtained by structural alignment of 23S rRNA.
